# Traditional medicinal plants used for the treatment of diabetes in rural and urban areas of Dhaka, Bangladesh – an ethnobotanical survey

**DOI:** 10.1186/1746-4269-9-43

**Published:** 2013-06-24

**Authors:** Soeren Ocvirk, Martin Kistler, Shusmita Khan, Shamim Hayder Talukder, Hans Hauner

**Affiliations:** 1 Else Kroener-Fresenius-Center for Nutritional Medicine, Klinikum rechts der Isar, 81675 Munich and ZIEL – Research Center for Nutrition and Food Sciences, Technische Universität München, 85350, Freising-Weihenstephan, Germany; 2 Eminence, 3/6 Asad Avenue, Dhaka 1207, Bangladesh

**Keywords:** Ethnobotanical survey, Diabetes mellitus, Traditional medicinal plants, Evidence-based medicine, Bangladesh

## Abstract

**Background:**

The usage of medicinal plants is traditionally rooted in Bangladesh and still an essential part of public healthcare. Recently, a dramatically increasing prevalence brought diabetes mellitus and its therapy to the focus of public health interests in Bangladesh. We conducted an ethnobotanical survey to identify the traditional medicinal plants being used to treat diabetes in Bangladesh and to critically assess their anti-diabetic potentials with focus on evidence-based criteria.

**Methods:**

In an ethnobotanical survey in defined rural and urban areas 63 randomly chosen individuals (health professionals, diabetic patients), identified to use traditional medicinal plants to treat diabetes, were interviewed in a structured manner about their administration or use of plants for treating diabetes.

**Results:**

In total 37 medicinal plants belonging to 25 families were reported as being used for the treatment of diabetes in Bangladesh. The most frequently mentioned plants were *Coccinia indica*, *Azadirachta indica*, *Trigonella foenum-graecum*, *Syzygium cumini*, *Terminalia chebula*, *Ficus racemosa*, *Momordica charantia*, *Swietenia mahagoni*.

**Conclusion:**

Traditional medicinal plants are commonly used in Bangladesh to treat diabetes. The available data regarding the anti-diabetic activity of the detected plants is not sufficient to adequately evaluate or recommend their use. Clinical intervention studies are required to provide evidence for a safe and effective use of the identified plants in the treatment of diabetes.

## Introduction

Bangladesh features a sub-tropical climate and low-lying landmass largely adjacent to extensive river deltas. The country comprises very fertile soils and is home to some rare ecosystems such as the Sundarbans mangrove forests. Given the fertile plains and high population density, the indigenous vegetation has mostly given way to cropland and extensive cultivation. Today, almost 60% of the landmass is used for farming, which is a global maximum value. However, originally large parts of Bangladesh featured tropical forests and marshy jungle with highly biodiverse flora - being also an excellent source for medicinal plants.

The Bangladeshi traditional medicine is a unique conglomerate of different ethnomedical influences. Due to the geographic location and sociocultural characteristics of the country, it involves traditionally rooted elements influenced by local indigenous people and close-by Indian Ayurveda and Unani medicine [1,2]. Given its inexpensive, easily accessible and well-established health services, the use of traditional medicine is an integral part of public health services in Bangladesh with its providers being deeply embedded within the local community [3-5]. Recent data suggest that the utilization of traditional medicine health services in Bangladesh is widespread [6] and plays a crucial role in providing health care for poor people, people in rural areas and for tribal people [4,7-13].

In the context of using traditional medicinal plants for treating diabetes, extensive screening has been performed in many ethnomedical systems within the Indian subcontinent [14,15]. However, in Bangladesh the traditional medicinal plants that are used for the treatment of diabetes have not yet been studied in great detail. Therefore, these herbal remedies are important objects of research, especially in context of the virtually exploding prevalence of diabetes mellitus in Bangladesh. Although diabetes is more prevalent in urban areas [16,17], in rural communities prevalence rates for diabetes rose from 2.3% to 6.8% in between 1999 to 2004 [18].

A recent survey in Bangladesh demonstrated that in slum areas, 86% of female and 78% of male diabetic patients use either inadequate medical treatment or none [19]. In non-slum areas only 34% of female and male diabetic patients undergo adequate medical treatment [19] raising the question, whether herbal remedies of the traditional Bangladeshi medicine may offer a safe, effective and reasonable alternative therapy for diabetes. To address this question, an identification of the plants being used in Bangladesh for the treatment of diabetes is essential. We conducted an ethnobotanical survey in defined rural and urban areas of Bangladesh to document and evaluate which plants are used for the treatment of diabetes.

## Material and methods

### Study area

The study was performed in both an urban district of Dhaka, as well as a rural region adjoining to the city. The urban part was conducted in Dhaka, which is the capital of Bangladesh and has an area of 304 km^2^. According to the 2008 estimate of the Bangladesh Bureau of Statistics, Dhaka has a metropolitan population of about 12.8 m inhabitants, of which most are of Bengali origin. Although the direct biodiversity in the city is obviously quite low, Dhaka - with its offer of labor and infrastructure - causes a continuous migration of new residents from all over Bangladesh. This leads to a diverse background of the inhabitants and was a major reason for conducting the survey in several *thanas* (subdistricts) in Dhaka on the professional informant side. For the interviews with diabetic patients we focused predominantly on a previously recruited cohort residing in the Mirpur subdistrict of Dhaka. The rural part of the survey was conducted in Manikganj, which is a part of Dhaka division. It is bounded by the Dhaka district on the east and south and bordered by the Jamuna and Padma River. The Manikganj district has an area of 1379 km^2^ and a population of 1.3 m inhabitants. Being a former subdivision of the Dhaka district, it features a rural environment with smaller towns and a lower population density than the urban areas of Dhaka.

### Ethnobotanical data collection and type of data collected

This study adhered to the research guidelines and ethical protocols of the Technical University of Munich. The aim of the study was to qualitatively identify traditional medicinal plants known and accessible by the Bangladeshi population. For this purpose, a dialogue with local political and health service authorities was initiated for authorization as well as support. To obtain the most valuable information in a reasonable sample size, interviews of key informants were performed. Altogether 63 interviews were conducted, of which 29 were attributed to rural and 34 to urban regions. To cover a diverse spectrum of informants, different informant groups were defined for participating in the study. From these informant groups, key informants were randomly chosen out of a pool of knowledgeable persons (Table 1). It is noteworthy that the sample size of informants participating in this study was limited and not representative for the study groups or regional distribution, so that quantitative conclusions are not feasible. However, to screen the data obtained in this study the overall frequency of citation of plants was assessed and is indicated in the results (Table 2).

**Table 1:** Sample size and demographic data of key informants

**Informant group**	**No of persons (urban/rural)**	**Gender (female/male)**	**Age**	**Professional experience**
Diabetic patients	9/9	6/12	53 (35–68)	-
Traditional healers (*Kabiraj*)	5/5	1/9	59 (25–82)	26 (2–50)
Indigenous medicine companies ^a^	5/-	0/5	50 (25–66)	8 (3–14)
Private indigenous healing centers	5/5	0/10	42 (25–57)	11 (1–23)
Indigenous doctors ^b^	5/5	1/9	42 (30–65)	8 (1–25)
Allopathic doctors	5/5	2/8	45 (39–52)	9 (4–20)

Average and range of age in years; average and range of professional experience of health practitioners in years; ^a^ = Representatives from indigenous medicine companies; ^b^ = Indigenous doctors passed from Unani and Ayurvedic Medical College and hospitals.

**Table 2:** List of medicinal plants used in traditional medicine for the treatment of diabetes in Bangladesh

**Botanical name (Voucher specimen ID)**	**Family**	**Local Name**	**Plant parts used**	**Stage of maturity**	**Frequency of citation**
*Achyranthes aspera* L. (BD-01)	Amaranthaceae	Upat Lengra	Root, whole plant	M	0.85
*Adiantum capillus-veneris* L. (BD-02)	Adiantaceae	Hanglapudi, Gobalelota	Seed	M	0.85
*Allium sativum* L. (BD-03)	Amaryllidaceae	Rôsun	Root, whole plant	M	1.69
*Andrographis paniculata* Wall. ex Nees (BD-04)	Acanthaceae	Kālmegh	Leaf, whole plant	M	0.85
*Asparagus racemosus* L. (BD-05)	Asparagaceae	Sotomuli	Root	M, F	1.69
*Azadirachta indica* A. Juss. (BD-06)	Meliaceae	Neem	Bark, leaf, seed	M, F	8.47
*Bunium persicum* Bois. (BD-07)	Apiaceae	Kalo Jeera	Seed, whole plant	M	1.69
*Centella asiatica* L. (BD-08)	Apiaceae	Thankuni	Leaf	M	0.85
*Coccinia indica* W.&A. (BD-09)	Cucurbitaceae	Kundri, Telachuka	Fruit, leaf, root, whole plant	M, F, Pm	16.95
*Cynodon dactylon* (L.) Pers. (BD-10)	Poaceae	Durba	Leaf, whole plant	M, F	1.69
*Datura stramonium* L. (BD-11)	Solanaceae	Dhotura	Seed	M	0.85
*Eclipta alba* L. (BD-12)	Asteraceae	Bringoraj, Kalokeshi	Leaf	M	0.85
*Ficus benghalensis* L. (BD-13)	Moraceae	Bot, Kathali Pata Bot	Leaf	M, F	1.69
*Ficus racemosa* L. (BD-14)	Moraceae	Joiggidumur	Bark, fruit	M, Pm	4.24
*Gymnema sylvestre* R. Br. (BD-15)	Asclepiadaceae	Medhasingi, Gorshar	Whole plant	M	0.85
*Heliotropium indicum* L. (BD-16)	Boraginaceae	Hatisur	Leaf	M	0.85
*Hemidesmus indicus* L. R. Br. (BD-17)	Apocynaceae	Anantomul	Root	F	0.85
*Lagerstroemia speciosa* (L.) Pers. (BD-18)	Lythraceae	Jarul	Leaf	M	2.54
*Mangifera indica* L. (BD-19)	Anacardiaceae	Aam	Seed	M	0.85
*Mimosa pudica* L. (BD-20)	Fabaceae	Lojjaboti, Sada Lojjaboti	Whole plant	M	0.85
*Momordica charantia* L. (BD-21)	Cucurbitaceae	Kôrola	Fruit, leaf, whole plant	M, F	4.24
*Musa sapientum* L. (BD-22)	Musaceae	Kôla	Fruit	M	0.85
*Ocimum sanctum* L. (BD-23)	Lamiaceae	Krisno Tulshi, Kalo Tulshi	Whole plant	M, F	0.85
*Phyllanthus emblica* L. (BD-24)	Phyllanthaceae	Amloki	Fruit, seed, whole plant	M, F	3.39
*Swertia chirata* L. (BD-25)	Gentianaceae	Chirota	Root	-	0.85
*Swietenia mahagoni* Jacq. (BD-26)	Meliaceae	Mahogany	Seed	M, F	4.24
*Syzygium cumini* (L.) Skeels (BD-27)	Myrtaceae	Jam	Leaf, seed	M, F	7.63
*Tamarindus indica* L. (BD-28)	Fabaceae	Tetul	Seed	M	1.69
*Terminalia arjuna* W.&A. (BD-29)	Combretaceae	Arjun	Seed	M	0.85
*Terminalia bellirica* L. (BD-30)	Combretaceae	Bohera, Jonglee Bohera	Seed	M, F	3.39
*Terminalia chebula* Retz. (BD-31)	Combretaceae	Horituki	Seed	M, F	5.08
*Tinospora cordifolia* Hook. F. & Thoms. (BD-32)	Menispermaceae	Gulancha lota	Bark, leaf, root, whole plant	M	3.39
*Trigonella foenum-graecum* L. (BD-33)	Fabaceae	Methi	Seed, whole plant	M, F	8.47
*Vernonia anthelmintica* Willd. (BD-34)	Asteraceae	Somraj	Whole plant	M	0.85
*Vinca rosea* L. (BD-35)	Apocynaceae	Golapi Noyontara	Leaf	F	0.85
*Vitex negundo* L. (BD-36)	Lamiaceae	Nirgundi, Nishinda, Samalu	Leaf	M	0.85
*Withania somnifera* (L.) Dunal (BD-37)	Solanaceae	Aswagandha	Leaf, root, whole plant	M, F	2.54

Alphabetically listed with plant parts used (listed alphabetically); stage of maturity of plant when being used (M = mature, F = fresh, Pm = premature); frequency of citation by informants.

Interviews were conducted in the Bengali language and based on a semi-structured question form with answers recorded. Informed consent was obtained from the participants for the publication of this report. The questionnaire was designed to gather information on social status and education of the informant, general knowledge about diabetes (and its diagnosis), access to allopathic medicine, and medicinal plants used in the therapy of diabetes. Formulations of plants were not included in this survey. Every key informant was interviewed once. Medicinal plants being mentioned by the informant were recorded with local names and photographed. Whenever possible, informants were asked to show or collect the plants they use for the treatment of diabetes. The documented plants and samples were dried, stored and identified with the help of a botanist.

### Data analysis

The frequency of citation was calculated to assess the incidence of one particular plant species used for the treatment of diabetes in relation to the overall citations for all plants. The frequency of citation for a plant species was calculated as follows: Frequency of citation for a particular species = (Number of citations for that particular species/Number of all citations for all species)*100. For the most mentioned medicinal plants of the survey, a literature search was performed with special focus on diabetes-related clinical data.

## Results

The 63 conducted key informant interviews of the ethnobotanical survey revealed 37 different plants that were mentioned by informants for anti-diabetic treatment individually or combined with other plants (Table 2). Regarding the overall frequency of citation, the most cited plants were *Coccinia indica*, *Azadirachta indica*, *Syzygium cumini*, *Trigonella foenum-graecum*, *Terminalia chebula*, *Ficus racemosa*, *Momordica charantia* and *Swietenia mahagoni*, suggesting a prominent role of these plants in the herbal treatment of diabetes.

**Figure 1:**
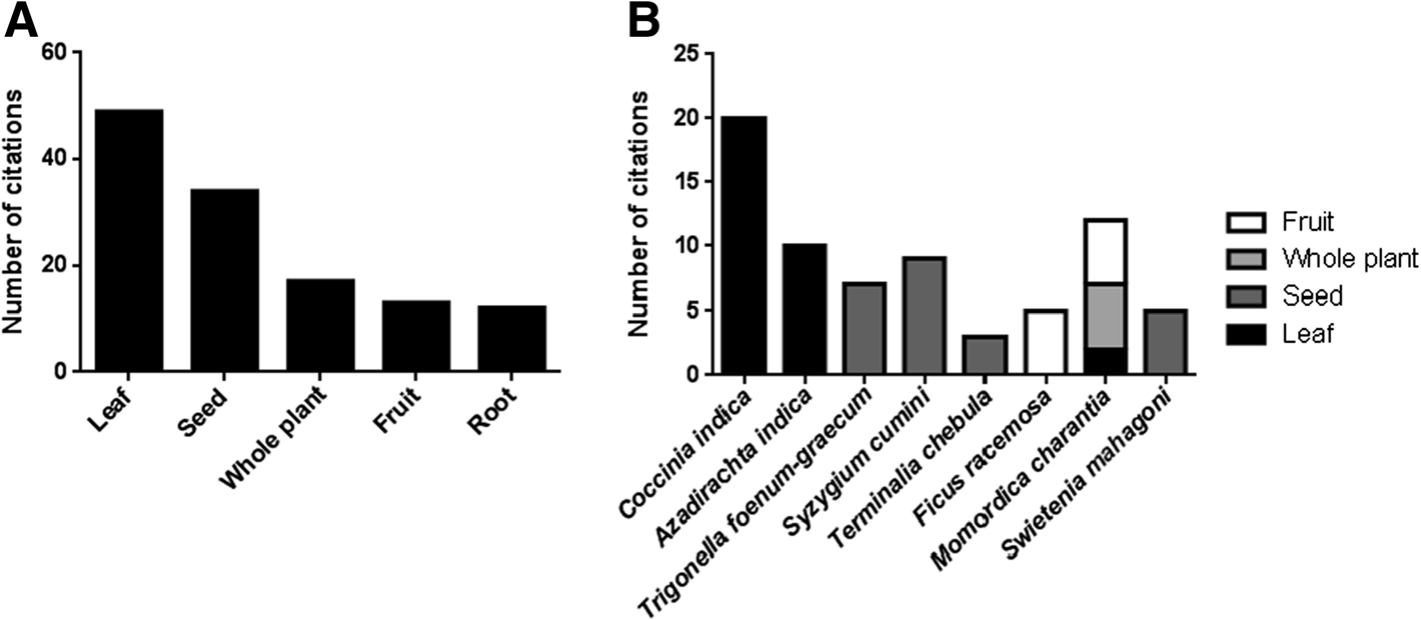
**Parts used for the treatment of diabetes from all plants (A) and the most frequently mentioned plants (B).** Multiple answers possible, some informants did not mention any specific plant parts.

Leaves and seeds at a defined stage of maturity were most frequently cited to be used for treatment or preparation of the traditional medicine (Figure 1A). Interestingly, plant parts used from the most frequently mentioned plants were consistent with the exception of *Momordica charantia* (Figure 1B)*.*

**Figure 2:**
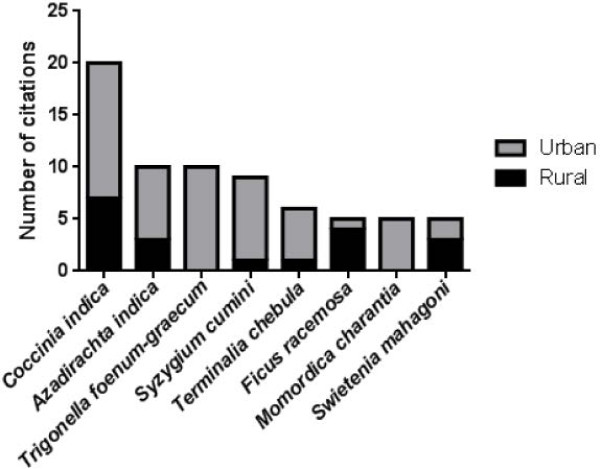
Regional distribution of the most frequently mentioned plants.

To receive an impression of regional availability of the top-mentioned plants, the number of citations in urban and rural areas was compared. Though only a few more informants were interviewed in urban areas (Table 1), the number of citations by urban informants prevails considerably for most plants (Figure 2). *Trigonella foenum-graecum* and *Momordica charantia* were exclusively mentioned by urban informants.

**Figure 3:**
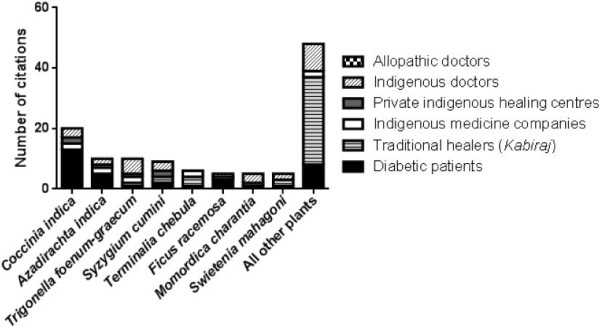
Citation of most frequently mentioned plants related to informant groups.

Finally, the most frequently mentioned plants were analyzed regarding times mentioned by the relevant informant groups for herbal treatment of diabetes. Admittedly quantitative comparisons between the groups were not feasible due to overall low numbers of informants and the high number of diabetic patients outnumbering all other groups. However, all top-mentioned plants were known by a minimum of three informant groups, suggesting a broad propagation of these plants within the different diabetes-related groups (Figure 3). The most mentioned medicinal plant, *Coccinia indica*, was also most prevalently used in the diabetic patients group highlighting its prominent role in herbal treatment of diabetes in the study area. In contrast, *Coccinia indica* was not cited by the traditional healer (*Kabiraj*) informant group. Interestingly, most of the not top-mentioned plants were cited by the *Kabiraj* informant group (Figure 3).

## Discussion

Overall, the survey revealed 37 medicinal plants belonging to 25 families that are used to treat diabetes in Bangladesh. Due to limited sample size, we focused this discussion on the most frequently mentioned plants.

Regarding all identified plants, leaves and seeds were the major plant parts used, which is in agreement with other studies [12,20,21]. The collection and processing of leaves and seeds is easy [21], and does not damage the plant substantially as compared to the collection of roots or the whole plant [22,23]. Leaves and seeds may contain or accumulate the pharmacologically active agents of plants. For example, these were reported for the seeds of *Trigonella foenum-graecum*[24,25] and *Syzygium cumini*[26] and the leaves of *Coccinia indica*[27-29] and *Azadirachta indica*[30].

A recent survey recorded plant parts similar to our study for *Coccinia indica*, *Azadirachta indica*, *Syzygium cumini*, *Trigonella foenum-graecum*, *Terminalia chebula* and *Momordica charantia* used in the treatment of diabetes by traditional health practitioners in Bangladesh [12]. In contrast to our study, *Kadir et al.* mentioned bark and root of *Ficus racemosa* to be used as medicinal plant component. Although most studies show anti-diabetic properties for the bark of *Ficus racemosa*[31,32], anti-diabetic agents were also isolated from its fruits [33]. Interestingly, *Swietenia mahagoni* was not reported as a medicinal plant used for the treatment of diabetes in Bangladesh. Since *Swietenia mahagoni* is not indigenous to Bangladesh, it may not be a common traditional medicinal plant for this area. However, it was cited by indigenous doctors (Ayurveda/Unani) as well as traditional *Kabiraj* healers in this survey. Recent reports emphasize the hypoglycemic and anti-oxidant activity of *Swietenia mahagoni* bark and seed extracts [34,35].

Regarding the regional distribution of plants, the most top-mentioned plants seem to be similarly available in urban and rural regions. Higher availability of plants in urban regions may reflect the role of Dhaka as socio-economic and commercial center of the country. *Trigonella foenum-graecum* and *Momordica charantia* were only mentioned by urban informants, suggesting a general low local availability of these plants in the rural surroundings of Dhaka. However, *Trigonella foenum-graecum*[24] and *Momordica charantia*[36,37] are widely available at local markets in Bangladesh.

*Coccinia indica* was mentioned by one third of all informants for being used as an anti-diabetic agent. In Bangladesh, *Coccinia indica* is well-known as an ayurvedic medicinal plant and is used for the treatment of diabetes [12,27,28]. It seems to be largely available in the area of Dhaka, since it was mentioned by most informants from the diabetic patients group. Leaves of *Coccinia indica* showed hypoglycemic effects in several animal studies [29,36,38,39] and small human intervention trials [28,40,41]. Though showing promising preliminary results, the anti-diabetic efficacy of *Coccinia indica* is not convincing due to the lack of solid evidence from extensive clinical intervention studies.

*Momordica charantia* is an edible vegetable commonly known as bitter gourd. It is widely available in Bangladesh and also well-known as an agent with several anti-diabetic effects [36,37,42,43]. Numerous studies revealed anti-hyperglycemic effects for its fruits in experimental animal studies of induced diabetes [36,37,44-46], but also the leaves, stem and seeds were reported to be used for anti-diabetic treatment [12]. Conflicting results were reported by small clinical trials; only modest hypoglycemic effects less distinct than for metformin were shown in type 2 diabetes mellitus patients [47], and no effect on the levels of plasma insulin and glucose was detectable in obese men [48], revealing the inconsistent outcomes for *Momordica charantia* regarding clinical trials [49].

Several small preliminary clinical studies investigating the effect of seed extracts of *Trigonella foenum-graecum* revealed a significantly reduced insulin resistance [50] and improved fasting and postprandial blood glucose levels [51,52] in diabetic patients. Isolated compounds of fenugreek, when administered in addition to sulfonylureas, also showed an improved anti-diabetic action compared to sulfonylureas alone in diabetic patients [53]. Since there are relatively many, but rather small human clinical trials available for fenugreek, it is one of the most interesting candidate plants for effective and safe anti-diabetic therapy.

*Azadirachta indica* is a common medicinal plant for tribal people in Bangladesh [12,13]. Anti-hyperglycemic effects in normal or diabetes-induced animal models were shown for leaf extracts of *Azadirachta indica*[30,39,54,55]. Seed extracts had significant hypoglycemic activity in a small cohort of type 2 diabetes patients [56].

For *Ficus racemosa*, few animal studies report blood glucose lowering activity [31-33,57]. In a preliminary clinical trial, treatment of type 2 diabetic patients with bark extracts of *Ficus racemosa* resulted in significantly reduced blood glucose levels and increased serum insulin levels [58].

Anti-diabetic effects were shown for *Terminalia chebula*[59-63] and *Syzygium cumini*[26,64,65] in normal or diabetes-induced animal models, but not in healthy individuals [66].

## Conclusion

The available clinical data suggesting anti-diabetic activity of plants identified in this survey is limited. Most of the clinical studies lacked sufficient sample size, randomized controlled study design or revealed only low anti-diabetic efficacy following the treatment with plants. In this context, it is also questionable to what extent the numerous anti-diabetic effects of plants and their extracts found in experimental animal and *in vitro* studies can be extrapolated to human settings. Out of the identified 37 plants used for the treatment of diabetes in Bangladesh only a few were shown to eventually exert anti-diabetic activity in clinical studies.

We henceforth propose to focus future research on the conduction of high-quality clinical studies while concentrating on those plants which show the most promising anti-diabetic efficacy in already performed clinical studies. In this context, it is also of particular interest to include safety issues and to study dose-dependent relationships. There are several other plants identified in this study of potential interest as preliminary data from animal or *in vitro* studies may indicate some anti-diabetic activity. But this has to be further investigated by clinical trials meeting the requirements of evidence-based medicine [67]. Such studies are of enormous public health interest as they may offer an evidence-based and safe use of non-expensive plant-derived medications against the growing epidemic of diabetes, particularly for low-income countries such as Bangladesh.

## Competing interests

The authors declare that they have no competing interest to disclose.

## Authors’ contributions

SO and MK designed and carried out the survey, analyzed the data and drafted the manuscript. SK recruited the informants, managed the field work and carried out the survey. SHT participated in designing the study and was contact person to administration authorities. HH participated in designing the study, data analysis and revising the manuscript. All authors read and approved the final manuscript.
